# Locating the Inexhaustible: Material, Medium, and Ambient Information

**DOI:** 10.3389/fpsyg.2020.00447

**Published:** 2020-03-13

**Authors:** Tetsushi Nonaka

**Affiliations:** Graduate School of Human Development and Environment, Kobe University, Kobe, Japan

**Keywords:** medium, ambient information, exploratory activity, reservoir computing (RC), tensegrity

## Abstract

The fundamental difference between the enactive approach and Gibson’s ecological approach lies in the view toward our shared environment. For Varela et al. (1991), a pregiven environment that exists “out there” is incompatible with the worlds enacted by various histories of life. For Gibson (1979/2015), the environment with its unlimited possibilities that exists out there offers many ways of life. Drawing on the recent empirical studies on the mechanical basis of information and pattern formation in a wide range of fields, this paper illustrates a principle regarding how pattern and change that are formed in an environmental medium, under certain conditions, could serve as the reservoir of information that makes available a variety of opportunities for perception. The second part of this paper offers a discussion about how the consideration of the materials that make up the terrestrial environment—the particles in the atmosphere and the textured surfaces—led Gibson to replace the concept of “space” with the notion of “medium” that allows for the open-ended activities of perception. Finally, I argue that given due consideration of the ambient information available in the medium, the apparent incompatibility between the world independent of the perceiver that exist out there and the worlds enacted by various histories of life could be resolved.

“Get rid, thoughtful Reader, of the Ockhamistic prejudice of political partizenship that in thought, in being, and in the development the indefinite is due to a degeneration from a primary state of perfect definiteness. The truth is rather on the side of the scholastic realists that the unsettled is the primal state, and that definiteness and determinateness, the two poles of settledness, are, in the large, approximations, developmentally, epistemologically, and metaphysically.”

—Peirce (1934, CP 6.348)

## The Many and the Reality

On August 22, 1970, Gibson wrote a letter to his colleague at Cornell University, a philosopher Norman Malcolm (Nonken, 2008, p. 288):

Dear Norman,

I meant that “the same stimulus array” will always afford the same perceptual experience insofar as it carries the same variables of structural information (Gibson, 1966).

In the case of your example, I would argue that structural sequence of sounds may in fact be music, 18th century music, Mozart, badly played Mozart, a sonata, etc. All of them are in the structure of the sounds. When a listener “hears” one rather than another, he does not detect a difference in the structure for the different perception, he only abstracts different features of the available structure. I do not mean that he detects different structures in each case. Structure, in sound and light, is inexhaustibly rich.

This is one case of perceiving as, only one.
Yours
Jimmy

The original context of the discussion between Malcolm and Gibson that led to the above Gibson’s response is unknown. However, the point that Gibson is making here referring to his previous work (Gibson, 1966, p. 248) is clear. Gibson argues that the multiplicity of perceptions of the same sequence of sounds derives from the richness of the available structure of the sounds in the air. Perception of sounds in the environment is direct, in the sense that it is the act of detecting the information from the available structure of sounds. However, at the same time, what is perceived would not be fixed like an automatic response to a stimulus. This is simply because the structure of sounds in the air is so rich that different invariant features can be selectively picked up from the same available structure. A music critic may be able to tell that a sequence of sounds is 18th century music, composition by Mozart, badly played Mozart, and a sonata, by picking up the subtle relations between notes with certain frequencies and how they unfold over time at different temporal scales (from vibratos to recapitulations in sonatas) in the air. On the other hand, a 5-year-old child who has never been exposed to classical music may not be able to distinguish the subtle difference in the style of playing Mozart, but only notice a certain orderliness of the temporal structure of the auditory event. Yet, there is still a possibility that, in the future, this same child will be able to discriminate a subtle combination of an enormous number of variables from another combination just like the music critique. This possibility is not entirely groundless, but is based on the fact that the available information in the structure of sound in the medium is inherently rich, with full of higher-order patterns and changes in a complex hierarchy of inter-nested levels of parts and subparts. “Structure, in sound and light, is inexhaustibly rich” even in the man-made artificial arrays like music.

At the first blush, what Gibson says plainly in this letter may not seem controversial. After all, no one would disagree with the fact that different people, or the same person on different occasions, sometimes pay attention to different features of the same thing in the environment. People do get better at discriminating something, find different use-values of the same thing, and take advantages of the different offerings of the environment depending on different situations and on different histories of life. But when Gibson went on to argue that what exists out there in the environment itself provides the basis of the multiplicity of perceptual experiences, Gibson’s remark might sound unsettling to representationalists and enactivists alike.

Traditionally, there has been a strong motivation to regard the incompatibility of multiple perceptual experiences as evidence that the world that exists “out there” is not perceived directly. Addressing this point, Smith (2009) wrote as follows: “our commonsense perceptual space, for example, has a Euclidean structure. The space of the physicist has another, quite different structure. And it may well be that the perceptual spaces of mice, of spiders, of clams, have other structures again. Not all of these structures can be true of space as it is in itself. Hence, the argument goes, our (and the mouse’s, and the spider’s) perceptual space are mere ‘mental representations.’ And what goes for space holds for other features of the manifold environments of perception, too—so that it is as if each species lives in its own special world (p. 128).” In order to reconcile “many” perceptual experiences with a “single” reality, the epistemic mediators such as mental representations are thus introduced. Thereby, we are left with another problem, that of how the environment and the mental representations can be matched and reintegrated.

Varela et al. (1991) maintained that both Gibson’s ecological approach and their enactive approach “deny the representationist view of perception in favor of the idea that perception is perceptually guided action (p. 203).”^1^ But at the same time, unlike Gibson, they also denied the idea that the objective world is known to us. In their influential book, Varela et al. (1991) proposed “as a name the term enactive to emphasize the growing conviction that cognition is not the representation of a pregiven world by a pregiven mind but is rather the enactment of a world and a mind on the basis of a history of the variety of actions that a being in the world performs (p. 9).” The name “enactive,” for them, derived from the conviction that “a pregiven world” and the worlds enacted “on a basis of a history of the variety of actions that a being in the world performs” are not compatible. This point was repeatedly emphasized in the aforementioned book, often in the context of criticism against Gibson’s ecological approach: “whereas Gibson claims that the environment is independent, we claim that it is enacted (by histories of coupling)…. From the fact, however, that there is a mutuality between animal and environment—or in our terms the two are structurally coupled—it simply does not follow that the act of perceiving is direct in the Gibsonian sense of “responding” or “resonating” to optical invariants (Varela et al., 1991, p. 204).”

The fundamental difference between Varela, Thompson, and Rosch’s enactive approach and Gibson’s ecological approach lies in the view toward our shared environment. Gibson emphasized that the environment offers many ways of life. “There are all kinds of nutrients in the world and all sorts of ways of getting food … all kinds of locomotion that the environment makes possible …. But, for all we know, there may be many offerings of the environment that have not been taken advantage of, that is, niches not yet occupied (Gibson, 1979/2015, p. 121).” Thereby, in Gibson’s ecological approach to perception, the theory of two worlds, in any form, is rejected. “There is only one environment, although it contains many observers with limitless opportunities for them to live in it (Gibson, 1979/2015, p. 129).” By contrast, Varela et al. (1991) did not acknowledge the rich possibilities offered by the environment, but attributed the different experiences by different agents to intrinsic factors and their corresponding worlds that are not “out there,” which is apparent in statements such as follows: “According to traditional wisdom, the environment in which organisms evolve and that they come to know is given, fixed, and unique. Here, again we find the idea that organisms are basically parachuted into a pregiven environment. …The key point, …, is that the species brings forth and specifies its own domain of problems to be solved by satisficing; this domain does not exist ‘out there’ in an environment that acts as a landing pad for organisms that somehow drop or parachute into the world. … what we describe as environmental regularities are not external features … (Varela et al., 1991, p. 198).” Without questioning the traditional characterization of the real environment as something that fixes what organisms come to know, they found the environment that exists “out there” simply incompatible with “the worlds enacted by various histories of structural coupling (Varela et al., 1991, p. 217).” This conviction further led them to abandon the idea of the world as independent and extrinsic. The crucial step taken here was the decision not to “retain the notion of an independent, pregiven environment but let it fade into the background in favor of so-called intrinsic factors (Varela et al., 1991, p. 198).” But, what if the environment that exists out there is not something that determines what is perceived by a perceiver, but proves to be itself sufficiently rich to provide the perceiver with open-ended possibilities of further exploration? As Gibson said in his letter to Malcolm, what if the variation found in perceptual experiences is not just the expression of the worlds enacted by various histories of life, but also an expression of the richness of the structure in ambient energy arrays that lies open to further scrutiny?

Of course, the latter Gibson’s view does not stand or fall on the basis of logical considerations, but is a matter for empirical inquiry. Two issues stand out for empirical inquiry: Where is the information for perception? Is the information sufficiently rich for the act of perception to be open-ended? Drawing on the recent empirical studies on the mechanical basis of information and pattern formation in a wide range of fields—mechanobiology, soft robotics, and sensory ecology—this paper illustrates a principle how pattern and change that are formed in an environmental medium, under certain conditions, could serve as the reservoir of information that makes available the open-ended opportunities for perception. Then, I shall discuss how the explicit consideration of the materials of the terrestrial environment—the particles in the atmosphere and the textured surfaces—led Gibson to replace the concept of “space” with the notion of “medium” that allows for the open-ended activities of perception. Finally, I argue that given due consideration of the ambient information available in the medium, the apparent incompatibility between the world independent of the perceiver that exist out there and the worlds enacted by various histories of life could be resolved.

## Information in Liquid

**FIGURE 1:**
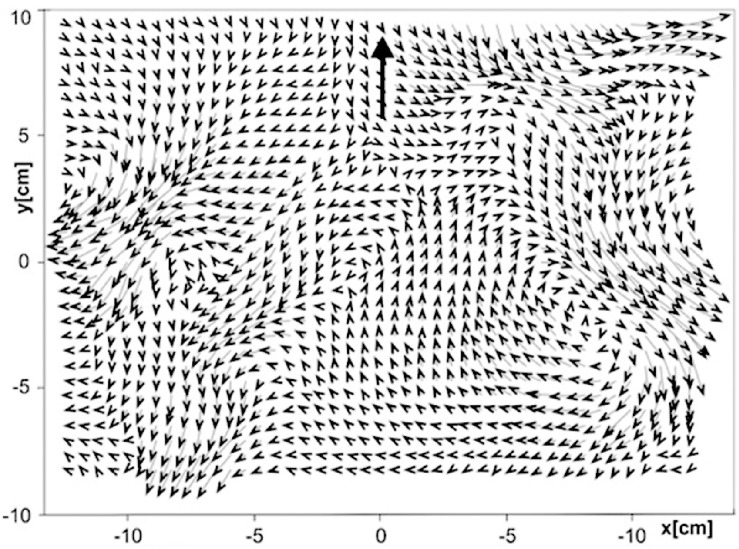
Water velocity 60 s after an 86 mm long fish (*Lepomis gibbosus*) passed the area. Bold arrow indicates swimming direction. Adapted from Hanke (2014). Copyright 2014 by Springer-Verlag.

As an illustration for the pattern and change that exist independent of perceivers that are potentially informative about the eventful world, let us first consider the case of so-called hydrodynamic perception—mechanosensing of water movements by aquatic animals. Most aquatic animals have developed perceptual systems to discriminate water disturbance (Hanke and Bleckmann, 2004). These water disturbances arise from a variety of sources such as other animals including predators, prey, conspecifics, inanimate objects and events, and the swimming movements of the perceiving animal itself (Hanke, 2014). For example, water disturbances caused by swimming fish of different species have been shown to have different wake signatures, which can last for several minutes (Figure 1). These water movements provide valuable sources of information for piscivorous predators at a distance not only about the presence of a fish of suitable size but also about the species or the swimming style of the fish that have passed by at an earlier point in time (Hanke and Bleckmann, 2004). A recent study showed that the flow structures caused by the fast-starting fish consisted of multiple jets that contain directional information, which are suited to provide aquatic predators not only with information on the presence of a fish of suitable size, but also on the direction of its escape (Niesterok and Hanke, 2013). Harbor seals—piscivorous mammals—are known to use their vibrissae to haptically discriminate the water movements left behind by prey or predator, and perceive the motion path, size and shape of the object that caused the trail (Hanke, 2014).

A point worth emphasizing is the fact that although the informative patterns of water movement are there to be perceived by an animal, there are many reasons that the animal may not attend to the information. The harbor seal running away from the white shark may not attend to a pattern of water movement that specify the presence of salmon that can be preyed upon. Near the surface of clear water during daytime the animal may attend to optical information without taking advantage of hydrodynamic information. The flow patterns that are informative about various aquatic events exist out there in the water. However, at the same time, the patterns of water movement do not fix what is perceived by perceiving animals. Instead, the ambient water makes available the open-ended opportunities for selectively picking up different features of the flow pattern depending on various life histories of perceiving animals. Hydrodynamic perception is possible provided the following two conditions are met: (1) the water movements are sufficiently sensitive to differentiate different source events, and (2) the perceiver has the skill and sufficient resolution to detect the difference between different water movements.

Quite independently from hydrodynamic perception research, the computational neuroscientist Maass has arrived at some similar viewpoints that the perturbed states of the medium could potentially make available the reservoir of information from which different task-specific information could be extracted. Maass et al. (2002) identified that the key challenge to understanding perception is to find the right conceptual framework that could explain how animals detect in real-time the equivalent state from the continuously changing stimulation which may never repeat. In tackling this challenge, Maass took seriously the material environment of neurons—the fact that “the neurons in our brain are embedded into an artificial sea-environment, the salty aqueous extracellular fluid which surrounds the neurons in our brain (Maass, 2002, p. 33).” Using the metaphor of liquid, Maass et al. (2002) wrote as follows:

… consider a series of transient perturbations caused in an excitable medium (see Holden et al., 1991), for example, a liquid, by a sequence of external disturbances (inputs) such as wind, sound, or sequences of pebbles dropped into the liquid…the perturbed state of the liquid, at any moment in time, represents present as well as past inputs, potentially providing the information needed for an analysis of various dynamic aspects of the environment. In order for such a liquid to serve as a source of salient information about present and past stimuli without relying on stable states, the perturbations must be sensitive to saliently different inputs… (Maass et al., 2002, pp. 2533–2534).

**FIGURE 2:**
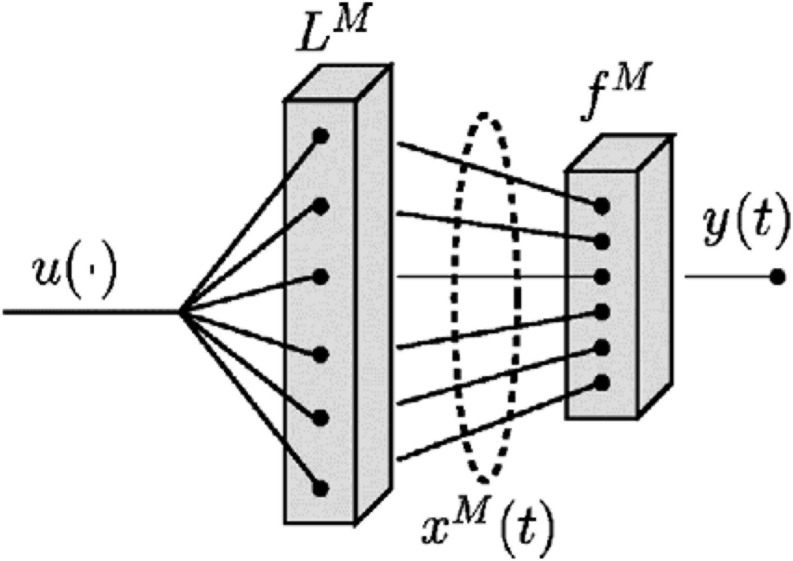
The architecture of a liquid state machine *M*. A time series *u*(⋅) is injected as input into the liquid filter *L*^*M*^, creating at time *t* the liquid state *x*^*M*^(*t*), which is transformed by a memoryless readout map *f*^*M*^ to generate an output *y*(*t*) (Maass et al., 2002). Adapted from Maass et al. (2002). Copyright 2002 by Massachusetts Institute of Technology.

Using perturbations caused in a medium as informational resources, Maass et al. (2002) proposed a computational model called the Liquid State Machine, which maps some function of time (e.g., an aspect of the environment that changes continuously) onto other functions of time (e.g., continuous adjustments of action in relation to the changing aspect of the environment) in real-time. Although the framework has been applied to modeling computation in neural microcircuits, the implications of the liquid state machine are general and can be applied to different material systems. Like the Turing machine which has universal power for off-line computing on discrete inputs, liquid state machines are supported by a rigorous mathematical framework that guarantees, under idealized conditions, universal power for real-time computation with fading memory on continuous functions of time (Maass et al., 2002). In terms of architecture, a liquid state machine *M* consists of the following two components: The first component is a medium called a liquid filter *L*^*M*^ which generates, at every time *t*, perturbed “liquid state” *X*^*M*^(*t*) in response to a preceding continuous sequence of disturbances *u*(⋅) (Figure 2). A liquid filter has the time-continuous property of fading memory whose state depends on the disturbances from some finite time window into the past, just as the perturbed states of the sea water lasts for some finite amount of time after a fish has passed by. A liquid filter needs not be customized for a specific task, just as sea water is pregiven and not customized for a specific task of each aquatic animal. The second component of a liquid state machine *M* is a memoryless readout map *f*^*M*^ that transforms, at every time *t*, the current liquid state *X*^*M*^(*t*) (which reflects the past as well as the current events because of the fading memory property of the liquid filter) into task-specific output *y*(*t*). In other words, a readout map *f*^*M*^ extracts the task-specific information from the reservoir of information available in the current liquid state, and continuously adjusts the output in relation to the task goal. Depending on the task, the readout can generate an invariant response quite independent from high-dimensional transient states of the liquid, by learning to define the task-relevant equivalence classes for the dynamic liquid states (Maass et al., 2002). Moreover, it is possible to add multiple readouts to a single liquid filter so that each readout extracts different task-specific information from the rich liquid state in such a way to support completely different, multiple real-time controls in parallel.

A liquid state machine is a time-invariant filter with fading memory that maps input stream onto an output stream. It has the universal power for real-time computing on perturbations regardless of specific implementation or structure, provided that the following two macroscopic properties are satisfied: a separation property and an approximation property (Maass et al., 2002). A separation property addresses the amount of separation between the trajectories of perturbed states of the liquid that are resulting from two different input disturbances (i.e., the difference between the wave patterns caused by different sequences of disturbances). An approximation property addresses the capability of distinguishing and transforming different perturbed states of the liquid into given target outputs (Maass et al., 2002). Whereas a separation property depends largely on the material characteristics of the liquid, an approximation property depends largely on the adaptability of the readout mechanism to the required task (Maass et al., 2002). Theoretically, the performance of liquid state machines improves with any improvement in their separation or approximation property (Maass et al., 2002).

The conceptual framework of a liquid state machine was later unified with so-called Echo State Networks—a machine learning approach independently developed by Jaeger that shares the fundamental operating principle (Jaeger and Haas, 2004)—under the overall label of *reservoir computing*. The framework of reservoir computing suggests that the reservoir (i.e., a medium such as a liquid filter in liquid state machines) that exists independent of the task-specific readouts, if it has rich and diverse enough dynamics to differentiate the different sources of disturbances, could make available the opportunities for real-time, task-specific control of the medium-readout system. In reservoir computing, the only task of the reservoir is to have its dynamic state perturbed by some event (Seoane, 2019). In doing so, through its non-linear, convoluted dynamics, the reservoir is picking up the event and projecting it into the high-dimensional space that consists of various possible dynamic configurations of the reservoir, which could potentially render relevant features from the event more easily separable. Provided that the separation property is satisfied, the implementation of the reservoir can be quite arbitrary—be it spider web sensitive to mechanical disturbances or a recurrent circuit of integrate-and-fire neurons with rich recurrent dynamics. This suggests the possibility that an arbitrary material system could be “found” and used as the reservoir in the course of evolution or development (as opposed to being “determined” by animals), from which information about different aspects of the eventful environment could be extracted.

## The Body as a Reservoir

**FIGURE 3:**
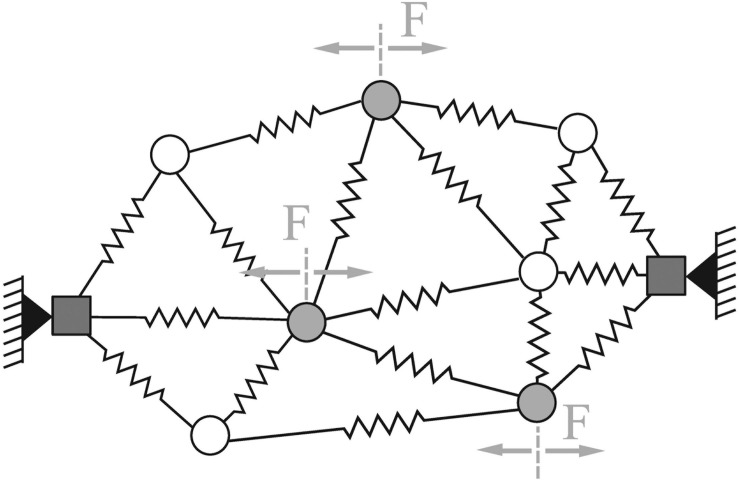
Schematic example of a generic mass-spring network. The mass points are connected by non-linear springs. The square masses are fixed in order to hold the network in place. The gray mass points are randomly chosen inputs nodes, which receive the input in form of horizontal forces scaled by randomly initiated weights (Hauser et al., 2011). Adapted from Hauser et al. (2011). Copyright 2011 by Springer-Verlag.

In engineering, structures with high degrees of dynamical coupling are known to be difficult to control, because these couplings tend to produce undesirable non-linear interactions that are difficult to predict (Rieffel et al., 2009). But what if there’s a possibility that these structures rife with dynamical coupling can be harnessed not as something whose movement is to be governed by a commander, but as a medium which provides the reservoir of information? Based on the framework of a liquid state machine, Hauser et al. (2011) used a simple generic model of physical body based on mass-spring systems to implement a liquid filter in a simulation experiment, in which the system learns the complex mapping of the movements of the end-effector of a robot arm on a horizontal plane to the joint torques required to produce the movements (i.e., inverse dynamics). They randomly positioned 30 mass points in a two-dimensional plane, which were then connected by 78 non-linear springs with randomly chosen heterogeneous properties (Hauser et al., 2011). Two sets of randomly chosen six mass points received linearly scaled horizontal disturbances *F* derived from the target *x* and *y* position in the plane, respectively, whose scaling factors were again randomly selected and fixed (Figure 3). Just like spider web, the entire mass-spring network responded to these disturbances in real-time by changing the 78 spring lengths, which were the 78-dimensional “liquid states” of the body. The linear readout of the system was defined as the weighted sum of continuously changing 78 spring lengths whose weights were adapted to the corresponding target torque in the learning process. After learning, the mass-spring networks successfully performed the complex mapping of the target movement of the end effector to the torque time series with extremely high accuracy (Hauser et al., 2011). When no mass-spring body was available and linear regression was applied on the raw input signals (i.e., *x* and *y* positions of the end effector) instead of the 78 spring lengths, the system was not able to adjust the momentary torque according to the target position. Interestingly, even when the mass-spring body was available, when all the spring properties were made homogeneous, the performance of the system severely deteriorated, implying that diversity is an important material property of the body that allows real-time guidance of movement (Hauser et al., 2011).

In a subsequent experiment, Nakajima et al. (2013) used an actual soft robotic arm, inspired the anatomy of an octopus, to control the movement of the arm in a closed-loop manner in such a way to embed non-linear limit cycles (e.g., the Van del Pol limit cycle). Using the similar mass-spring network as Hauser et al. (2011), they fed the output generated by the system (weighted sum of all the spring lengths of the arm) back into the system as a motor command for the next timestep which generated the rotation movement of the arm (Nakajima et al., 2013). And then the spring lengths of the arm perturbed by the self-movement were in turn used as resources to adjust the torque that controls the movement of the arm. In the experiment, they trained the linear readout to obtain the optimal readout weights to emulate a non-linear limit cycle, and then switch the motor command to the system output generated by the trained readout weights. They found that the motor output of the arm exhibited almost a complete fit with the target trajectory for some types of limit cycles, which was robust against external noise over an extended period of time (Nakajima et al., 2013). These studies implied that a complex structure rife with dynamical coupling can be harnessed as a medium that provides rich resources for controlling the *movements* of the system of which such structure is a part. However, it should be noted that whether and how task-specific information could be actively sought after in the reservoir so as to control the system’s *encounters* with a cluttered environment still remains to be seen.

The manner in which reservoir computing is conventionally discussed is as a model of computation to perform complex mappings from input sequences to output sequences (e.g., to emulate dynamical systems). In this context, the main concern has been the information provided by the states of a medium perturbed by *passive* input. We shall now turn to *active* exploration that brings about disturbances in a medium in such a way to isolate informative structures. Gibson’s classic paper on active touch offered a stark illustration of the contrast between a passive receptive channel of sensory input and an active system that hunt for information (Gibson, 1962). The haptic apparatus of animals incorporates mechanoreceptors that are distributed across different parts and organs of the body—in and below the skin, the ligaments connecting the joints between the movable bones, and the muscles and the tendons. It is notable that what all these parts and organs have in common is that they can be moved and deformed, actively as well as passively (Gibson, 1966, p. 108). Recently, Turvey and Fonseca (2014) hypothesized that interconnected structural hierarchies composed of tensionally prestressed networks of our bodies that span from the macroscale to the microscale—from muscles, tendons, and other connective tissues to various micro-elastic structures such as a network of collagen fibers—constitute the medium for the haptic sense organs of animals. Because the form of any structure, whether a vortex flow of water or a living tissue, is determined through a dynamic interplay of physical forces, the distinct pattern of forces characteristic of a mechanical disturbance may convey a physical form of information that constrain perception and behavior of an agent (Ingber, 2005). Like the air being the medium for sound, odor, and reverberating flux of light, despite being on the other side of the skin, Turvey and Fonseca (2014) argued, the presence of isometric tension distributed throughout all levels of interconnected, multiscale networks make available the opportunities for an active perceiver to spontaneously perturb the tensionally integrated system in such a way to isolate the invariant patterns that specify the source of mechanical disturbances.

For the haptic perception of the properties of hand-held objects, evidence to date suggests that different properties of a held object are independently perceivable (for reviews, see Turvey and Carello, 2011). Wielding an object exerts reactive forces and torques on skin, muscles, ligaments, and tendons. From this array of deformation, perceivers are shown to be able to selectively pick up information that specifies properties such as length, width, and crude shape of the hand-held object. The manner of active exploration has also been shown to differ systematically as a function of the particular property to be attended to. For example, when perceiving how far a hand-held object extends from hand (i.e., the object’s length), wielding movement about the wrist joint is typically observed (Carello and Turvey, 2017). Thereby, a rotation point defined in the wrist is always at a fixed distance from an object held in the hand, enabling the invariant rotational inertia specific to the length of the object to be isolated over the time-varying motions of the limb and accompanying deformation of tissues. By contrast, when perceiving the width of a hand-held object such as a tennis racket, twisting movements about the longitudinal axis of the object is typically observed, allowing the invariant rotational inertia specific to the width to be isolated over the time-varying motions of the limb (Arzamarski et al., 2010). Moreover, a variety of available opportunities for exploration is related to the fact that the same property of the object is perceivable by means of different neuromuscular patterns of movement. Silva et al. (2009) reported that a patient with stroke-induced motor impairment who had restricted movements of the wrist was nevertheless able to perceive the length of the rod secured to the hand with an elastic band with the same accuracy and reliability as individuals without movement disorders. This patient wielded the rod not about the wrist but about the longitudinal axis of the arm through the shoulder joint, which was kept at a fixed distance from the end of the rod. Despite the very different kinematics and transformation of tissue deformation arrays, the wielding movement apparently contributed to separating off the same invariant as did the wrist movement (Silva et al., 2009).

**FIGURE 4:**
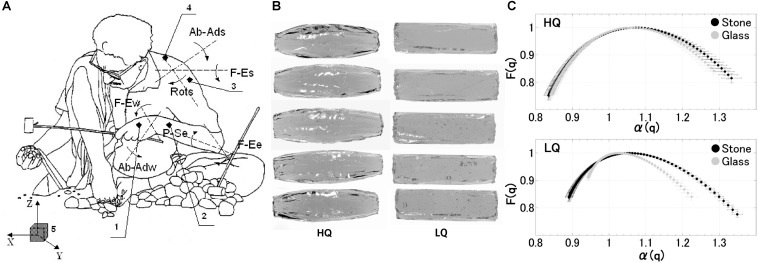
**(A)** Typical posture and movement of craftsmen during stone bead production. **(B)** Examples of ellipsoidal glass beads produced by expert (HQ) and non-expert (LQ) craftsman. **(C)** Singularity spectrum *f*(α(*q*)) (–1.4 ≤ *q* ≤ 3) estimated for expert (HQ) and non-expert (LQ) craftsmen in the conditions using carnelian stone (black spheres) and glass (gray spheres) as raw materials. The vertical and horizontal bars are standard errors of the means for *f*(*q*) and α(*q*), respectively, of multiple realizations of each condition (Nonaka and Bril, 2014). Adapted from Nonaka and Bril (2014). Copyright 2014 by American Psychological Association.

In recent years, there has been a growing interest in the problem concerning the transformation and variation produced by active exploratory movement to separate the information about aspects of the environment relevant to the task at hand (Nonaka, 2019). In general, exploration requires fluctuations, and fluctuations increase in time. A growing body of evidence indicates that the fluctuations in exploratory behaviors exhibit the property of superdiffusion, where the fluctuation grows faster than normal diffusion governed by a Gaussian probability density function (e.g., Stephen et al., 2010; Viswanathan et al., 2011). Nonaka and Bril (2014) studied the exploratory movement of expert stone beads craftsmen in India who shape a bead by a series of hammer strikes on a stone held against the pointed tip of an iron bar (Figure 4). In the field experiment, the craftsmen shaped the ellipsoidal beads made of two different materials (carnelian stone—familiar material, and glass—unfamiliar, much more fragile material) in the studios they normally work. The use of the unfamiliar material must require an acute sensitivity to the properties of the material, where the finer the exploration, the better the probable outcome of the activities that follow. In the exploratory tapping movement of the craftsmen during the preparatory phase of the task, they found (a) the presence of long-range correlations where the variance of the displacement time series of the hand wielding the hammer grows superlinearly in time, and (b) underlying multiplicative interactions between fluctuations at different temporal scales indicated by the heterogeneity of scaling properties over time. When faced with the unfamiliar condition using unusual, fragile material, the exploratory hammer tapping movement of highly skilled experts who were able to cope with the situation exhibited a pronounced increase in the long-range temporal correlations. By contrast, the wielding behavior of less skilled experts—those who could not shape the glass beads—exhibited a significant loss of long-range correlations and reduced heterogeneity of scaling properties over time, which robustly discriminated the groups with different skill levels (Figure 4). Alterations in multiscale temporal structure of movement fluctuations were apparently associated with changes in the situation differently depending on the level of expertise (Nonaka and Bril, 2014).

The empirical evidence derived from this field experiment, albeit a special case of an unusually complex skill, may well serve for the purpose of constraining the possible accounts of active touch. Traditionally, corollary discharge from motor areas to somatosensory regions of the cortex has been considered to play a key role in active touch, which provides posterior parietal neurons with information on intended actions, allowing these neurons to compare planned and actual neural responses to tactile stimuli (Kandel et al., 2013, p. 524). Such an account focuses on sensory receptors and nervous system without reference to the architecture of the body where they are embedded. However, the problem expert craftsmen face is unlikely to be that of associating central and peripheral signals. The presence of non-linearity arising from multiplicative interaction across fluctuations at different timescales would greatly complicate such a process, with no simple correspondence between central motor commands, resulting movements and peripheral sensory feedback arising from them (Nonaka, 2019). Instead, the result is a much better fit to the alternative scenario of active touch that takes into account the medium for the haptic perceptual system (Turvey and Fonseca, 2014), in which efficacy of active touch depends on the tuning of the whole system including the multiscale tensile states of the body, the structures of which are transformed by exploratory behavior in such a way to differentiate the invariant patterns that specify the source of mechanical disturbances from all the other patterns that do *not* specify the source (Gibson, 1966, p. 55).

## The Medium

The notion of medium is the centerpiece underlying Gibson’s claim that the activity of perception is open-ended. Gibson used the term *medium* to refer to the material through which energy flows (e.g., wave propagation) and an animal travels, which is, after all, the dictionary meaning of the word. Gibson liked to contrast “space” with “medium.” “Space” is often regarded as an empty room that is blank and immaterial as opposed to the material objects. For example, the influential geographer Tuan (1977) wrote, “space means room (p. 51)” where material objects can be put in, and “space is experienced directly as having room in which to move (p. 12),” while “place and objects define space (p. 17).” By contrast, Gibson argued that what allows movement is not empty space but the gaseous atmosphere with specific material properties that offer low resistance to animal movement. The environment of animals consists of matter in the solid state and matter in a liquid or gaseous state, as well as the surfaces between them. Solid surfaces generally reflect rather than transmit light. They also generally prevent rather than permit locomotion. In contrast, through the liquid or gaseous regions of the environment, a detached solid body can move without much resistance. “It thus affords locomotion to an animate body. A gas or a liquid, then, is a medium for animal locomotion. Air is a better medium for locomotion than water because it offers less resistance (Gibson, 1979/2015, p. 12).” Gibson rightly recognized that the distinction between that which allows movement and that which does not has nothing to do with the empty/filled or material/immaterial dichotomy. Instead, this distinction is connected with the states of the surrounding material (e.g., solid, liquid, and gas). “Objects do not fill space, for there was no such thing as empty space to begin with. … The world was never a void. As for the medium, the region in which motion and locomotion can occur, where light can reverberate and surfaces can be illuminated, this might be called room but it is not space (Gibson, 1979/2015, p. 93).”

Not only allowing movement and locomotion, a terrestrial medium is a region in which light not only is transmitted but is scattered by particles in the atmosphere that is never perfectly transparent. Unlike mechanical waves mentioned in the example of hydrodynamic perception, electromagnetic waves such as light may occur in a vacuum as well as in a material medium. When light gets transmitted not in a vacuum but through the material medium of the atmosphere, the light is scattered by particles in the atmosphere, the amount of which depend on atmospheric conditions. This light is even more thoroughly scattered when it strikes the textured terrestrial surfaces. The scatter-reflected light is in turn reflected back from the particles in the atmosphere. Each new reflection further disperses the incident rays. The light thus finds its way into cluttered environment that are not open to the light source, “through chinks and crevices and into caverns (Gibson, 1979/2015, p. 44).” In semi-enclosed spaces the light continues to bounce back and forth at enormous velocity, and the reverberation of multiple-reflected light in a terrestrial medium reaches a steady state almost instantly. “This light can hardly be thought of as radiation now; it is illumination (Gibson, 1979/2015, p. 44).” Illumination is a fact of higher order than radiation. Many-times reflected light in a medium has a number of consequences important for visual perception. Chief among them is the fact of ambient light, that is, light that surrounds a point, any point, in the medium where an observer could be stationed (Gibson, 1979/2015, p. 45). The reverberating flux of light in the medium brings about the condition in which there is light coming to the point from all directions.

Although important for the act of visual perception, the distinction between the light which affords active exploration and the light which does not is rarely stated. There is experimental evidence that seeing the surfaces depends on the structure of the ambient optic array which has different intensities in different directions (Gibson et al., 1955). There is also evidence that the eye would be unable to focus in homogeneous ambient light (i.e., in the unusual case where light that surrounds a point of observation would not be different in different directions). If the light coming to the nodal point of the eye has no discontinuities of intensity in different directions, then it is impossible to accommodate your eyes (Gibson, 1982a, p. 92). In consequence, “the possessor of the eye could not fix it on anything, and the eye would drift aimlessly” (Gibson, 1979/2015, p. 47). When the light available to the eye is wholly undifferentiated, then you cannot actively explore the array, even though you may have a sensation of light. A vertebrate eye can extract information from ambient light only when the ocular system can accommodate, and for this the ambient light must be structured. It must constitute an optic array having an arrangement (Gibson, 1982a, p. 92).

In a terrestrial medium, radiant light becomes ambient light with full of higher-order patterns and changes in a complex hierarchy of inter-nested levels of structures and substructures. Ambient light is not to be confused with radiant light. Radiant light from the light source traveling in *space* itself does not carry informative structures about the surfaces of the environment. By contrast, radiant light traveling in the atmospheric *medium*, due to the multiple scattered reflections between surfaces of the particles in the air and those of the cluttered environment, results in ambient light where any point in the medium is structured by the light reflected from surfaces so that these characteristics are specified, which could render features of the substances, surfaces, places, things, and events potentially separable.

We are tempted to call the medium “space,” but the temptation should be resisted. For the medium, unlike space, permits a steady state of reverberating illumination to become established such that it contains information about surfaces and their substances. That is, there is an array at every point of observation and a changing array at every moving point of observation. The medium, as distinguished from space, allows compression waves from a mechanical event, sound, to reach all points of observation and also allows the diffusion field from a volatile substance, odor, to reach them (Gibson, 1979/2015, p. 216).

The notion of medium allows the distinction between potential and effective stimulation. We can now talk about *potentially* visible surfaces that could be looked at from some place in the medium where an animal might be, without making slightest reference about the actual stimulation of an eye and sensations of vision (Gibson, 1979/2015, p. 19). Radiant, acoustic, and chemical energy that are propagated through the medium provides the ambient sea of stimulus energy in which animals can move about. Instead of inquiring whether one model of inferring the causes of sensation aroused by stimuli is better than another, with the notion of medium we can now begin to study activity before sensations have been aroused by stimuli, an activity that orients the organs of perception and explores the sea of potential stimulation for the information external to the perceiver (Gibson, 1982b, p. 398). Unlike points in space defined by an arbitrary frame of reference, the ambient energy array surrounding each potential point of observation is unique (Gibson, 1979/2015, p. 14). As the observer moves from one point of observation to another, the optical array, the acoustic array, and the chemical array are transformed accordingly (Gibson, 1979/2015, p. 13). This provides the opportunities for an *active* observer to move in the medium to detect invariants underlying the transforming perspectives in the ambient array surrounding a moving point of observation.

The kind of information that may be obtained by exploration in the medium is fundamentally different from the kind of information available in a so-called visual image (e.g., a retinal image). For example, consider the case of distinguishing an obstacle from an opening in a cluttered environment. An obstacle affords collision. An opening affords passage. Both have a closed or nearly closed contour. The contour could be the edge of an obstacle such as a goblet, or could be the edge of an opening between a pair of faces as in the goblet-faces display described by Edgar Rubin. The way to tell the difference between an obstacle and an opening is inextricably tied to the manner of exploration by the perceiver in the medium: Loss (or gain) of structure outside a closed contour during the perceiver’s approach (or retreat) in the medium specifies an obstacle. Gain (or loss) of structure inside a closed contour during the perceiver’s approach (or retreat) in the medium specifies an opening (Gibson, 1979/2015, p. 219). As the perceiver come up to the obstacle it hides more and more of the vista, and as you come up to the opening it reveals more and more of the vista. A closed contour as such does not specify an obstacle or an opening in a cluttered environment. But deletion outside the occluding edge and accretion inside the occluding edge will distinguish the two. A certain manner of exploration in the medium results in a unique optical transformation that provides access to these invariants under transformation that specify an obstacle or an opening. Thereby, the perceiver tunes in on the invariant structure of the ambient optic array that underlies the changing perspective structure caused by her own exploratory movements (Gibson, 1979/2015). The whole flux of reverberating light pervading the medium is a potential stimulus which can be sampled at various points of observation in the medium, although it must be explored by embodied locomotor action of a perceiver. There are families and super-families of invariant information in a transforming ambient optic array at a moving point of observation in the medium, and by separating off such information, the perceiver in turn guides and controls locomotion, steers away from an obstacle, and enters an opening (Gibson, 1958/1998).

If we understand the notion of medium, I suggest, we come to an entirely new way of thinking about perception and behavior. The medium in which animals can move about (and in which objects can be moved about) is at the same time the medium for light, sound, and odor coming from sources in the environment. An enclosed medium can be “filled” with light, with sound, and even with odor. … As the observer moves from point to point, the optical information, the acoustic information, and the chemical information change accordingly. Each potential point of observation in the medium is unique in this respect. The notion of a medium, therefore, is not the same as the concept of space inasmuch as the points in space are not unique but equivalent to one another (Gibson, 1979/2015, pp. 13–14).

The fact is worth remembering that normal perception always involves the possibility of further exploration (of scrutinizing, of looking more carefully) whether or not the possibility is taken advantage of (Gibson, 1978). But, what makes us aware of the possibility of further exploration in the first place? What makes us aware of the layout of the environment in and out of sight? What makes it possible for animals to discover the potentially meaningful features of the environment that have not yet been taken advantage of? These questions share concerns regarding the same fundamental issue which cannot be resolved without restoring the following two terms: (1) an active perceiver that explores the environment, and (2) a reservoir of potential information about the environment that exists independent of the active perceiver. Without them, it would be impossible to disentangle a set of variables that are specific to the world out there (i.e., independent of the point of observation). With the notion of medium, at which Gibson arrived through the reconsideration of the materials that make up the terrestrial environment, we can locate the region in which information is sought after by an active perceiver, as well as the flowing array of energy that provides the opportunities for the activity of perceiving.

## Epilogue

Varela et al. (1991) viewed perception as follows: “perception consists in perceptually guided action. … the reference point for understanding perception is no longer a pregiven, perceiver-independent world but rather the sensorimotor structure of the perceiver (the way in which the nervous system links sensory and motor surfaces). This structure—the manner in which the perceiver is embodied—rather than some pregiven world determines how the perceiver can act and be modulated by environmental events (p. 173).” Yet, despite their characterization of perception as perceptually guided action, they did not mention the embodied act of perceiving—exploratory activity—that orients the organs of perception such as looking, listening, touching, tasting, and sniffing which involves muscular adjustments of organs to explore the rich structure of ambient energy arrays. What they also left out in their criticism against Gibson was the notion of medium. The lack of mention of these two notions in their book was probably not a coincidence. Failing to locate the ambient information available in a medium, Varela et al. (1991) did not have a means to clearly disentangle the existing information available to a perceiver from the information selectively picked up by the perceptual activity of the perceiver. Accordingly, they could not find where the embodied act of perceiving could literally *take place*. By virtue of not having a proper characterization of rich possibilities offered by the environment, Varela et al. (1991) were not able to talk about what perception is *after*. As a consequence, they could only talk about perceptually guided action, but not about what the activity of perception (e.g., the accommodation of the ocular system) is a constant function of (c.f., Holt, 1915).

Luminous, mechanical, or chemical energy is structured by the substantial environment and becomes ambient in the medium. The ambient sea of energy around each of us is usually very rich in what we call pattern and change, which is limitless in variables of higher order. The variables and co-variables and invariables of this stimulus environment are inexhaustible (Gibson, 1979/2015, p. 233). The environment, so considered, would consist of a reservoir of possible stimuli for both perception and action (Gibson, 1982c, p. 344). Taking into account the inexhaustible reservoir of information, what has been known tacitly is made explicit: The activity of perception is “open-ended,” and you can keep discovering new features and details about the environment by the act of scrutiny (Gibson, 1979/2015, p. 245).

… whether or not a potential stimulus becomes effective depends on the individual. It depends on the species to which he belongs, on the anatomy of the sense organs, the stage of maturation, the capacities for sense organ adjustment, the habits of attention, the activity in progress, and the possibilities of educating the attention of the individual (Gibson, 1982c, p. 346).

The present paper reviewed empirical evidence that supports the idea that the environment that exists out there is itself sufficiently rich to provide the perceiver with open-ended possibilities of further exploration. I believe that Varela et al. (1991) criticism against Gibson mentioned in the introduction was based on misunderstanding of Gibson’s “new” description of the indefinitely rich environment on which his approach was founded. The notion of the environment as a reservoir of limitless opportunities for both perception and action effectively eliminates any need to invoke the notion of *two worlds* or *many worlds* to rationalize the multiplicity of viable histories of structural coupling. With an adequate description of what the environment may offer to perception, the environment that exists out there and various viable streams of perceptual experience would turn out to be compatible. What the recognition of the environment with its unlimited possibilities brings to us is a theory of unlimited further discovery for perception, in which the apparent incompatibility between the many and the reality is resolved.

## Author Contributions

TN wrote the manuscript.

## Conflict of Interest

The authors declare that the research was conducted in the absence of any commercial or financial relationships that could be construed as a potential conflict of interest.

## Footnotes

1 In the present article, I deliberately chose to limit my discussion on ecological psychology and enactive theories to the original approaches proposed by Gibson (1979/2015) and Varela et al. (1991), respectively, for the purpose of comparison, although I’m aware that diverse views are present in both approaches (e.g., Di Paolo et al., 2018). A comprehensive review of the literature on both approaches is beyond the scope of the present article.
